# Effect of simulated acute bilateral severe conductive hearing loss on static balance function in healthy subjects: a prospective observational pilot study

**DOI:** 10.1007/s00405-023-07942-w

**Published:** 2023-03-31

**Authors:** Johanna Westermann-Lammers, Jawad Salameh, Christian Dobel, Orlando Guntinas-Lichius

**Affiliations:** Department of Otorhinolaryngology, Institute of Phoniatry/Pedaudiology, Jena University Hospital, Am Klinikum 1, 07747 Jena, Germany

**Keywords:** Sudden conductive hearing loss, Vestibular function, Computed posturography, Postural stability

## Abstract

**Purpose:**

Maintaining static balance is a process coordinated by central integration of visual, vestibular and somatosensory information. Whether or not hearing and spatial acoustic information contributes to the maintenance of static postural balance is unclear.

**Methods:**

A prospective observational pilot study was performed. Twenty-five normal hearing adults (68% female; 19–31 years) underwent a computerized dynamic posturography test battery including the Sensory Organization Test (SOT), the Motor Control Test (MCT), and the Adaptation Test (ADT). The balance tests were performed two times, in a randomized sequence without or with acute hearing loss. Earplugs (sound insulation 37 dB) or headphones with white noise (sound volume 75 dB) induced the conductive hearing loss. Hence, all participants passed through four sequences of the balance test battery. A repeated-measures analysis of variance (ANOVA) was used to analyze the results.

**Results:**

The ANOVA revealed no difference for any SOT and ADT subtest without hearing loss and simulated hearing loss (either earplugs or headphones; all *p* > 0.05). The ANOVA showed no longer latencies with simulated hearing loss compared to no hearing loss in both experiments with one exception: the reaction of the right foot during large forward translation was longer with hearing loss than without hearing loss in both experiments (*p = *0.025).

**Conclusions:**

Overall, a simulated acute conductive bilateral moderate or severe hearing loss did not disturb the static balance function in normal hearing younger adults in this first small pilot study.

**Supplementary Information:**

The online version contains supplementary material available at 10.1007/s00405-023-07942-w.

## Introduction

The continuous maintenance of static balance in upright standing is a complex process. The position of the human body relative to gravity and spatial surroundings is mainly sensed by central integration of visual, vestibular and somatosensory inputs [1]. The role of hearing and spatial acoustic information to maintain static postural balance is not completely understood. It seems to be obvious that auditory and vestibular systems function together, but their exact mechanism of interaction is not clear. Chronic hearing loss but also aging is associated with postural instability [2]. This might be the age-dependent result of the inner ear cell damage effecting both the hearing and vestibular organ [3]. The redundancy of the normal multisensory static balance system allows for a certain degree of compensation for loss of function of one of the involved systems (visual, vestibular and somatosensory, central integration) [4]. Postural dysfunction first becomes clinically visible if more than one of the components is highly damaged. Interestingly, the situation can be improved if one system is rehabilitated: hearing aids can improve static balance function in elderly with moderate to severe hearing loss [5]. The same can be seen in deaf patients receiving a cochlear implant. These patients often have postural dysfunction not obvious in daily life but only during forced postural perturbations. The reason is that these patients have an inner ear damage of both the cochlea and the vestibular system [6]. Hence, it is unproven, if chronic hearing loss could affect the postural control in a completely normal vestibular system.

In this regard, it is of interest to see if sudden acute hearing loss in younger patients affects postural stability. In a recent study by Horowitz et al., all but one static balance tests remained unchanged in young normal-hearing persons before and after the ears were plugged with earplugs [7]. Surprisingly, based on the significant deterioration in one subtest (when the somatosensory input was disrupted), they broadly concluded that acute conductive hearing loss has a negative effect on balance. Actually, the deterioration was only seen when another component, the somatosensory input was completely blocked. Therefore, we repeated and extended the experiment, a) using two acute hearing loss settings (earplugs and then headphones with white noise), b) randomizing the sequence of testing without and with acute hearing loss to avoid a learning effect, and c) by a standardized static balance test battery consisting of the Sensory Organization Test (SOT), the Motor Control Test (MCT) and the Adaptation Test (ADT) protocols, all in normal hearing healthy probands.

## Materials and methods

### Study design and setting

This prospective observational pilot study was carried out by the Department of Otorhinolaryngology, Jena University Hospital, Jena, Germany. Approval for the study was obtained through the local ethics committee (No. 2021–2248-BO) and written informed consent was obtained from all study participants. The study included two experiments using two different methods to simulate the acute hearing loss in 25 healthy subjects. Earplugs were used in the first experiment and headphones with white noise in the second experiment. All participants performed the static balance test without and with simulated hearing loss. To rule out a learning effect, the sequence, without and with simulated hearing loss, was randomized in each participant in both experiments [8]. The balance test after application of the earplugs or the headphones was performed without adaptation phase. The balance test started directly after application of the earplugs or headphones.

### Selection of the healthy participants

Only healthy, normal hearing subject with normal balance function were included. The participants received a pure tone audiogram (software evidENT3, Merz Medizintechnik, Reutlingen, Germany). The bone conduction hearing threshold at 0.5, 1, 2, and 4 kHz had to be below 20 dB. Unterberger stepping test and video head impulse test (software eHIT, Merz Medizintechnik, Reutlingen, Germany) had to be normal. Persons with a history of an ear or balance disease were excluded.

### Experimental setting 1: simulation of acute bilateral hearing loss with earplugs

Standard earplugs were used (uvex x-fit, SNR 37 dB, uvex Arbeitsschutz GmbH, Fürth, Germany) in both ears. The sound insulation value of these plugs is 37 dB. An example of a pure tone audiogram of a subject wearing the earplugs is shown in Supplement Fig. 1.

**Fig. 1:**
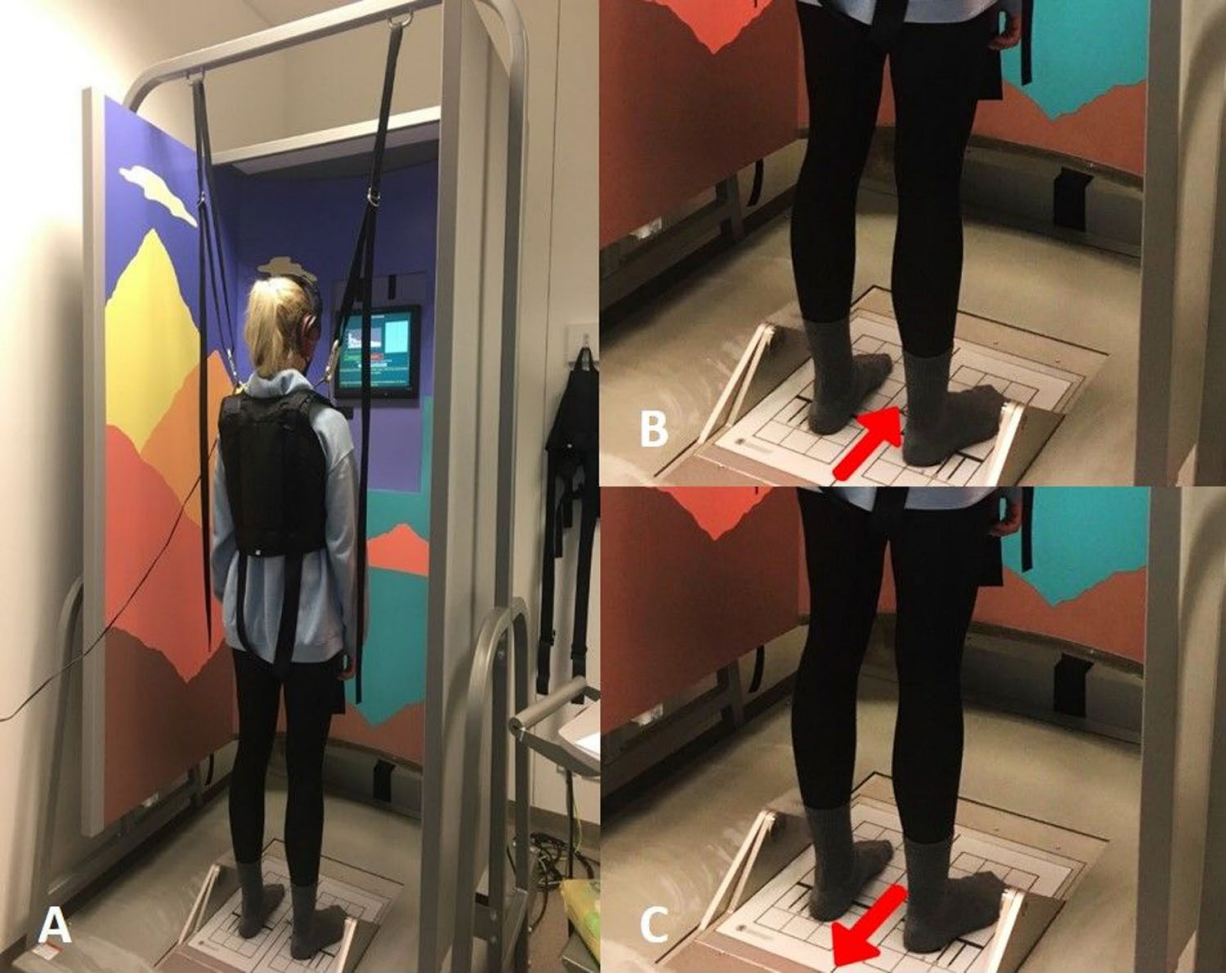
**A** Computerized static balance test system. The participant is wearing the headphones of experiment 2. **B** As an example, the setting of the Motor Control Test (MCT) is shown, here forward translation: **C** backward translation of the MCT

### Experimental setting 2: simulation of acute bilateral hearing loss with headphones and white noise

In this setting, the simulation was performed using headphones (Sennheiser HDA 280, Wedemark, Germany) and white noise with a sound volume of 75 dB. An example of a pure tone audiogram and a speech audiogram of a subject wearing the headphones with white noise is shown in Supplement Fig. 1.

### Static balance tests

A computerized dynamic posturography system (NeuroCom SMART EquiTest System; Natus Medical Incorporated, Clackamas, Oregon, USA) was used. The SMART EquiTest uses a computerized dual force plate system that allows real-time movement and center of gravity (COG) estimations based on pressure changes on the platform related to the participant’s height. The force sampling frequency was set at 100 Hz. The test battery consisted of the Sensory Organization Test (SOT), the Motor Control Test (MCT) and the Adaptation Test (ADT) protocols.

The SOT allows a separated or combined manipulation of visual, vestibular, and somatosensory feedback of the static balance system using six test conditions with eyes open/closed, fixed/moving surround, fixed/moving platform [9]. The subject’s postural sway was measured during three 20-s trials. For each condition equilibrium scores (from 0 = loss of balance to 100 = little if any shear force and perfect stability) were calculated by comparing the angular difference between the participant’s measured maximum anterior to posterior COG displacement to the theoretical sway stability limit of 12.5º. A composite score was calculated by averaging the scores from conditions 1 and 2; adding these 2 scores to the equilibrium scores from each trial of conditions 3, 4, 5, and 6.

The MCT measures the subject’s automatic postural reaction as a reaction to support unexpected surface translations in a standardized manner [9]: participants maintained their eyes open, and the surround remained stationary throughout the MCT. The MCT consisted of four conditions: backward and forward translations, with medium translation with a 1.8 º sway either for 300 ms or with large translation with a 3.2º sway for 400 ms, respectively.

The translations were scaled according to the person’s height. The durations were the same for everyone. Each translation started randomly after 1.5–2.5 s to prevent a foreseeability of the translation. As outcome measure of the MCT, the latency scores from the MCT were determined by calculation of the elapsed time in ms between onset of support surface translation until the point when the person actively resisted the induced sway.

The ADT measures motor reactions following abrupt platform rotations to the direction of toes up and down, with an amplitude of 8º and duration of 400 ms [10]. Participants performed five trials for each direction, and the mean of the trials and conditions was considered. The mean sway energy (ranging from 0 to 200) for toes up and down was measured. This energy is calculated based on the weighted sum of the root mean square of the velocity and acceleration of the anteroposterior center of pressure displacement. Lower scores reflect better adaptation on minimizing sway after the support surface rotation.

### Statistical analysis

Participants’ characteristics and outcome variables were analyzed with IBM SPSS statistics software (Version 23.0) for medical statistics. Data are presented as mean ± standard deviation (SD) if not otherwise indicated. Explorative statistics were used as no sufficient data on the effects of a simulated hearing loss on static balance were available. SOT, MCT, and ADT outcomes were compared using repeated-measures analysis of variance (ANOVA). The results 1) without and with simulated hearing loss, 2) between experiment 1 and 2, and 3) also interactions were compared. *p* values of 0.05 or less were considered significant.

## Results

### Study participants

Seventeen female and eight male participants were examined. The mean age was 22.4 ± 2.2 years (range 19–31). Mean body height was 1.7 ± 0.1 m (1.6–2.0). Mean body weight was 66.4 ± 12.5 kg (range 49–110). This resulted in a BMI of 22.0 ± 2.6 (range 19.1–31.5).

### Sensory Organization Test (SOT)

The highest (best) values were achieved in condition 1 (without earplugs: 94.9 ± 2.2; with earplugs: 94.6 ± 2.0; without headphones and white noise: 94.9 ± 1.9; with headphones and white noise: 95.4 ± 1.3; Table 1). The lowest (worst) results were seen for condition 5 (70.2 ± 9.0; 69.4 ± 10.2; 73.8 ± 6.8; 77.7 ± 6.2, respectively). The ANOVA revealed no difference for any SOT subtest without hearing loss and simulated hearing loss (either earplugs or headphones; all *p* > 0.05; Table 2). Nearly all results (exception: condition 1: *p = *0.168) were significantly better in experiment 2 than in experiment 1 (condition 2: *p = *0.019; condition 3: *p = *0.001; condition 4: *p = *0.011; condition 5: *p* < 0.001; condition 6: *p = *0.001; composite: *p* < 0.001). There was no significant interaction (all *p* > 0.05).

**Table 1:** Results of the static balance tests using earplugs or headphones with white noise to induce acute hearing loss

Parameter	Experiment 1: earplugs		Experiment 2: headphones	
	Without hearing loss	With hearing loss	Without hearing loss	With hearing loss
	Mean ± SD	Mean ± SD	Mean ± SD	Mean ± SD
Sensory Organization Test (SOT)				
Condition 1	94.9 ± 2.2	94.6 ± 2.0	94.9 ± 1.9	95.4 ± 1.3
Condition 2	92.7 ± 1.9	92.6 ± 2.2	93.3 ± 2.0	93.2 ± 2.1
Condition 3	91.9 ± 3.3	92.0 ± 3.2	93.7 ± 2.0	93.8 ± 2.1
Condition 4	90.0 ± 4.3	90.9 ± 3.2	91.9 ± 2.2	91.9 ± 2.7
Condition 5	70.2 ± 9.0	69.4 ± 10.2	73.8 ± 6.8	77.7 ± 6.2
Condition 6	78.2 ± 9.6	74.6 ± 12.1	83.1 ± 5.0	81.5 ± 5.3
Composite	84.3 ± 4.4	83.5 ± 5.1	86.8 ± 2.7	87.3 ± 2.7
Motor Control Test (MCT), latency (ms)				
Motor Control Test (MCT), latency (ms)				
Small backward left	132.9 ± 8.6	131.3 ± 9.0	130.8 ± 8.3	129.6 ± 6.9
Medium backward left	134.5 ± 12.3	133.0 ± 9.2	133.0 ± 9.8	132.5 ± 9.7
Large backward left	127.6 ± 10.5	128.8 ± 10.1	126.0 ± 11.2	128.4 ± 11.4
Small backward right	133.0 ± 9.7	130.9 ± 9.5	129.1 ± 9.0	130.9 ± 6.7
Medium backward right	131.9 ± 9.3	136.7 ± 25.4	131.0 ± 7.7	131.9 ± 9.3
Large backward right	128.6 ± 9.9	129.1 ± 10.2	129.1 ± 10.2	127.3 ± 11.2
Small forward left	125.7 ± 10.2	128.6 ± 10.3	127.1 ± 10.7	123.6 ± 8.4
Medium forward left	128.0 ± 11.5	131.0 ± 13.3	127.0 ± 18.7	127.5 ± 13.7
Large forward left	127.3 ± 11.2	128.2 ± 12.6	130.9 ± 12.3	134.6 ± 9.6
Small forward right	133.9 ± 19.4	131.5 ± 9.9	126.9 ± 12.5	129.2 ± 10.4
Medium forward right	127.1 ± 10.1	130.5 ± 13.2	126.2 ± 16.0	129.5 ± 17.7
Large forward right	125.7 ± 10.4	127.8 ± 13.5	131.3 ± 15.5	135.7 ± 15.3
Composite	130.14 ± 8.4	131.7 ± 9.0	130.8 ± 10.5	132.4 ± 7.9
Adaptation Test (ADT), sway energy				
Toes up 1	67.5 ± 14.0	73.3 ± 32.4	61.3 ± 14.7	60.2 ± 15.2
Toes up 2	63.6 ± 15.2	60.7 ± 18.6	56.7 ± 17.9	57.6 ± 12.4
Toes up 3	60.1 ± 13.3	62.3 ± 21.7	52.2 ± 12.2	52.4 ± 8.6
Toes up 4	52.6 ± 9.4	55.3 ± 14.4	51.6 ± 10.1	49.2 ± 7.0
Toes up 5	55.7 ± 10.5	58.6 ± 26.7	54.3 ± 11.6	46.9 ± 8.0
Toes down 1	52.6 ± 14.9	55.5 ± 14.7	49.4 ± 10.5	46.4 ± 9.2
Toes down 2	45.4 ± 10.9	45.2 ± 10.8	43.1 ± 10.6	41.3 ± 7.5
Toes down 3	42.2 ± 7.3	43.2 ± 11.6	40.8 ± 9.8	41.7 ± 12.9
Toes down 4	41.5 ± 7.6	42.7 ± 10.3	41.3 ± 12.5	38.6 ± 7.1
Toes down 5	42.4 ± 8.6	43.4 ± 16.0	39.3 ± 8.1	38.8 ± 8.3
Mean Tows up	60.0 ± 9.9	62.1 ± 20.4	55.2 ± 10.8	53.2 ± 7.7
Mean Tows down	44.8 ± 7.4	46.0 ± 11.5	42.8 ± 8.6	41.4 ± 8.1

*SD* standard deviation

**Table 2:** Repeated-measures analysis of variance (ANOVA) comparing the results a) without and with simulated hearing loss, and b) between experiment 1 and 2, and c) the interactions

Parameter	Difference without vs. with hearing loss	Difference experiment 1 vs. 2	Interaction
Sensory Organization Test (SOT)			
Condition 1	*F*(1, 24) = 0.065; *p = *0.801	*F*(1, 24) = 2.016; *p = *0.168	*F*(1, 24) = 0.797; *p = *0.381
Condition 2	*F*(1, 24) = 0.059; *p = *0.811	*F*(1, 24) = 6.367; ***p = *0.019**	*F*(1, 24) = 0.002; *p = *0.964
Condition 3	*F*(1, 24) = 0.027; *p = *0.870	*F*(1, 24) = 15.406; ***p = *0.001**	*F*(1, 24) = 0.006; *p = *0.939
Condition 4	*F*(1, 24) = 0.729; *p = *0.402	*F*(1, 24) = 7.558; ***p = *0.011**	*F*(1, 24) = 0.566; *p = *0.459
Condition 5	*F*(1, 24) = 1.514; *p = *0.230	*F*(1, 24) = 20.703; **p < 0.001**	*F*(1, 24) = 3.566; *p = *0.071
Condition 6	*F*(1, 24) = 3.090; *p = *0.092	*F*(1, 24) = 15.125; ***p = *0.001**	*F*(1, 24) = 0.454; *p = *0.507
Composite	*F*(1, 24) = 0.040; *p = *0.844	*F*(1, 24) = 31.474; p** < 0.001**	*F*(1, 24) = 1.344; *p = *0.258
Motor Control Test (MCT), latency (ms)			
Small backward left	*F*(1, 23) = 1.000; *p = *0.328	*F*(1, 23) = 2.620; *p = *0.119	*F*(1, 23) = 0.046; *p = *0.833
Medium backward left	*F*(1, 19) = 0.248; *p = *0.624	*F*(1, 19) = 0.521; *p = *0.479	*F*(1, 19) = 0.112; *p = *0.741
Large backward left	*F*(1, 24) = 1.699; *p = *0.205	*F*(1, 24) = 0.857; *p = *0.364	*F*(1, 24) = 0.302; *p = *0.588
Small backward right	*F*(1, 22) = 0.023; *p = *0.880	*F*(1, 22) = 2.314; *p = *0.142	*F*(1, 22) = 1.868; *p = *0.186
Medium backward right	*F*(1, 20) = 0.851; *p = *0.367	*F*(1, 20) = 1.132; *p = *0.300	*F*(1, 20) = 0.399; *p = *0.535
Large backward right	*F*(1, 21) = 0.417; *p = *0.525	*F*(1, 21) = 0.417; *p = *0.525	*F*(1, 21) = 0.665; *p = *0.424
Small forward left	*F*(1, 13) = 0.049; *p = *0.828	*F*(1, 13) = 0.513; *p = *0.486	*F*(1, 13) = 3.545; *p = *0.082
Medium forward left	*F*(1, 19) = 1.274; *p = *0.273	*F*(1, 19) = 0.719; *p = *0.407	*F*(1.19) = 0.629; *p = *0.437
Large forward left	*F*(1, 21) = 3.241; *p = *0.086	*F*(1, 21) = 7.219; ***p = *0.014**	*F*(1, 21) = 0.851; *p = *0.367
Small forward right	*F*(1, 12) = 0.000; *p = *1.000	*F*(1, 12) = 2.610; *p = *0.132	*F*(1, 12) = 0.602; *p = *0.453
Medium forward right	*F*(1, 20) = 2.642; *p = *0.120	*F*(1, 20) = 0.160; *p = *0.693	*F*(1, 20) = 0.000; *p = *1.000
Large forward right	*F*(1, 22) = 5.783; *p = *0.025	*F*(1, 22) = 7.114; *p = *0.014	*F*(1, 22) = 0.799; *p = *0.381
Composite	*F*(1, 13) = 1.722; *p = *0.212	*F*(1, 13) = 0.299; *p = *0.594	*F*(1, 13) = 0.000; *p = *1.000
Adaptation Test (ADT), sway energy			
Toes up 1	*F*(1, 23) = 0.220; *p = *0.643	*F*(1, 23) = 11.266; ***p = *0.003**	*F*(1, 23) = 1.255; *p = *0.274
Toes up 2	*F*(1, 24) = 0.309; *p = *0.584	*F*(1, 24) = 3.378; *p = *0.079	*F*(1, 24) = 0.397; *p = *0.535
Toes up 3	*F*(1, 24) = 0.249; *p = *0.623	*F*(1, 24) = 16.024; *p = ***0.001**	*F*(1, 24) = 0.256; *p = *0.617
Toes up 4	*F*(1, 24) = 0.007; *p = *0.934	*F*(1, 24) = 4.689; ***p = *0.041**	*F*(1, 24) = 2.454; *p = *0.130
Toes up 5	*F*(1, 24) = 0.950; *p = *0.339	*F*(1, 24) = 5.889; ***p = *0.023**	*F*(1, 24) = 2.863; *p = *0.104
Toes down 1	*F*(1, 24) = 0.000; *p = *0.993	*F*(1, 24) = 11.060; ***p = *0.003**	*F*(1, 24) = 2.625; *p = *0.118
Toes down 2	*F*(1, 24) = 0.994; *p = *0.329	*F*(1, 24) = 5.274; ***p = *0.031**	*F*(1, 24) = 0.612; *p = *0.442
Toes down 3	*F*(1, 24) = 0.312; *p = *0.581	*F*(1, 24) = 1.554; *p = *0.225	*F*(1, 24) = 0.002; *p = *0.961
Toes down 4	*F*(1, 24) = 0.648; *p = *0.429	*F*(1, 24) = 5.303; ***p = *0.030**	*F*(1, 24) = 2.079; *p = *0.162
Toes down 5	*F*(1, 24) = 0.036; *p = *0.852	*F*(1, 24) = 5.525; ***p = *0.027**	*F*(1, 24) = 0.176; *p = *0.678
Mean Tows up	*F*(1, 24) = 0.001; *p = *0.981	*F*(1, 24) = 14.520; ***p = *0.001**	*F*(1, 24) = 0.934; *p = *0.343
Mean Tows down	*F*(1, 24) = 0.011; *p = *0.916	*F*(1, 24) = 11.0; ***p = *0.003**	*F*(1, 24) = 1.789; *p = *0.194

Significant *p* values (*p* < 0.05) in bold

### Motor Control Test (MCT)

The results of the MCT are also presented in Table 1. The ANOVA revealed no longer latencies with simulated hearing loss compared to no hearing loss in both experiments with one exception: the reaction of the right foot during large forward translation was longer with hearing loss than without hearing loss in both experiments (*p = *0.025; Table 2). The comparison between experiment 1 and 2 showed that the latencies during large forward translation were longer in experiment 2 (*p = *0.014). There was no significant interaction (all *p* > 0.05).

### Adaption test (ADT)

The results of the ADT are also presented in Table 1. The ANOVA revealed no differences without and with simulated hearing loss in both experiments (Table 2). The comparison between experiment 1 and 2 showed differences. The participants needed less sway energy in experiment 2 in nearly all trials (toe up 1: *p = *0.003; toe up 3: *p = *0.001; toe up 4: *p = *0.041; toe up 5: *p = *0.02; toe up mean: *p = *0.001; toe down 1: *p = *0.003; toe down 2: *p = *0.031; toe down 4: *p = *0.030; toe down 5: *p = *0.027; toe down mean: *p = *0.003). There was no significant interaction (all *p* > 0.05).

## Discussion

Studies on the associations between acute or chronic hearing acuity and postural balance as well as between hearing acuity and falls are scarce and the results have been contradictory [11]. Some studies found only a minor or no association between chronic hearing loss and postural balance or falls [12, 13]. Some other studies found an association between hearing loss and postural control or falls [14]. A recent Finnish twin study revealed that people with chronic hearing loss have a higher risk for falls, which is partially explained by their poorer postural control [11]. The easiest explanation is that there may be a concomitant age-dependent dysfunction of both the cochlear and vestibular sense organs given their shared location within the inner ear. To our knowledge, this has not been shown yet, but decreased hearing might result in decreased auditory spatial information leading to balance disturbances even in older people with normal vestibular function. It might be that hearing aid use might improve static balance function [15]. A third explanation is that chronic hearing loss may also limit access to auditory cues that are needed for environmental awareness. Attentional resources are critical for maintaining postural control [16], and decrements in attentional and cognitive resources imposed by hearing loss may impair the maintenance of postural balance [14, 17].

To analyze the role of normal hearing on static balance in people with normal vestibular function, experiments like the present study simulating acute hearing loss not allowing any adaptation to the acute impairment are important. Such studies are even scarcer than studies with people with chronic hearing loss. Horowitz et al. used also earplugs (like in experiment 1 in the present study) to induce acute bilateral hearing loss in 20 normal hearing young adults [7]. They also used the SOT and the MCT as static balance tests. They saw only a significant deterioration in condition 4 of the SOT. The other SOT conditions and all MCT subtest showed no difference (It is irritating that Horowitz et al. nevertheless interpreted the results in a way that acute hearing loss affects postural stability). Hence, the present study mainly confirmed the results of Horowitz et al. and showed this in addition for the ADT: acute moderate hearing loss had no impact on static balance function. Furthermore, we showed that even severe acute hearing loss (experiment 2 with headphones and white noise) had no influence on static balance function. The only other study analyzing the effect of acute disturbed auditory acuity on static balance function is from [18]. The used a Nintendo Wii™ gaming console (i.e., no reference values for clinical setting are available) for static balance testing of postural sway. The tests were performed first without simulated hearing loss and then with ear defenders. Unfortunately, any information on these ear defenders is missing. Hence, the effect on hearing in the study by Kanegaonkar et al. remains unknown. Wearing these defenders in a normal room or a soundproof room showed no clear effect on the test results: only in the setting with a normal and with open eyes on foam (*p = *0.0495), in a soundproof room with open eyes on foam (*p = *0.0164), or with closed eyes on foam (*p = *0.0495), wearing ear defenders resulted on more postural sway. The *p* values are even marginal in two of these three setting. In all other four conditions, the defenders had no significant impact (all *p* > 0.05). Hence, also the work of Kanegaonkar et al. is more confirming than contradicting the present results. From a practical point of view, it can be concluded that acute hearing loss (for instance by a noise trauma at work) should not affect static balance. This could be important for occupations with high demands on static postural stability.

The present study was limited to normal hearing 19–31-year-old adults. The sample size was small. Hence, the results should be interpreted with caution. As a next step, we want to confirm the results in a larger cohort of young adults. Second, we want to repeat the study with older adults allowing the normal spectrum of hearing loss in such an older cohort. Furthermore, our focus is on static balance. That does not simply mean that acute bilateral moderate or severe hearing loss also has no influence on dynamic balance. When older adults (65–86 years) with no diagnosed hearing loss walk on a treadmill, ear plugging results in longer step length and step time [18]. However, ear plugging or white noise had no influence on the control of center of body mass. Overall, the authors interpret this in a way, that during a steady-state walking task, healthy older adults can maintain walking control without auditory feedback. However, it might be that temporal auditory cues provide locomotor feedback that becomes increasingly valuable as balance deteriorates with age. We interpret these results in context to the present finding as follows: auditory information or feedback signals, if at all, plays only a subordinate role for postural control in healthy younger adult, but might be relevant if one of the important information channels (visual, vestibular and somatosensory inputs) is altered, for instance, in older people.

## Conclusion

A large test battery consisting of the SOT, MCT, and ADT has shown that normal hearing adults between 19 and 31 years of age principally show an unaltered normal static balance during a simulated acute conductive moderate or severe bilateral hearing loss, at least in this first small pilot study. This suggest that hearing or spatial information does not play an important role to support static balance even during postural challenges in young adults.

## Supplementary Information

Below is the link to the electronic supplementary material.

Supplementary file1 (PPTX 121 KB)

## Data Availability

The datasets used and analysed during the current study are available from the corresponding author on reasonable request.
