# Supplementary material for: Effect of simulated acute bilateral severe conductive hearing loss on static balance function in healthy subjects: a prospective observational pilot study
Source: Eur Arch Otorhinolaryngol. 2023 Mar 31;280(7):3445–51. doi: 10.1007/s00405-023-07942-w (PMC10219876; doi:10.1007/s00405-023-07942-w)
Supplement: Supplementary file 1 — Supplementary file1 (PPTX 121 KB) [file 405_2023_7942_MOESM1_ESM.pptx]

## Slide 1
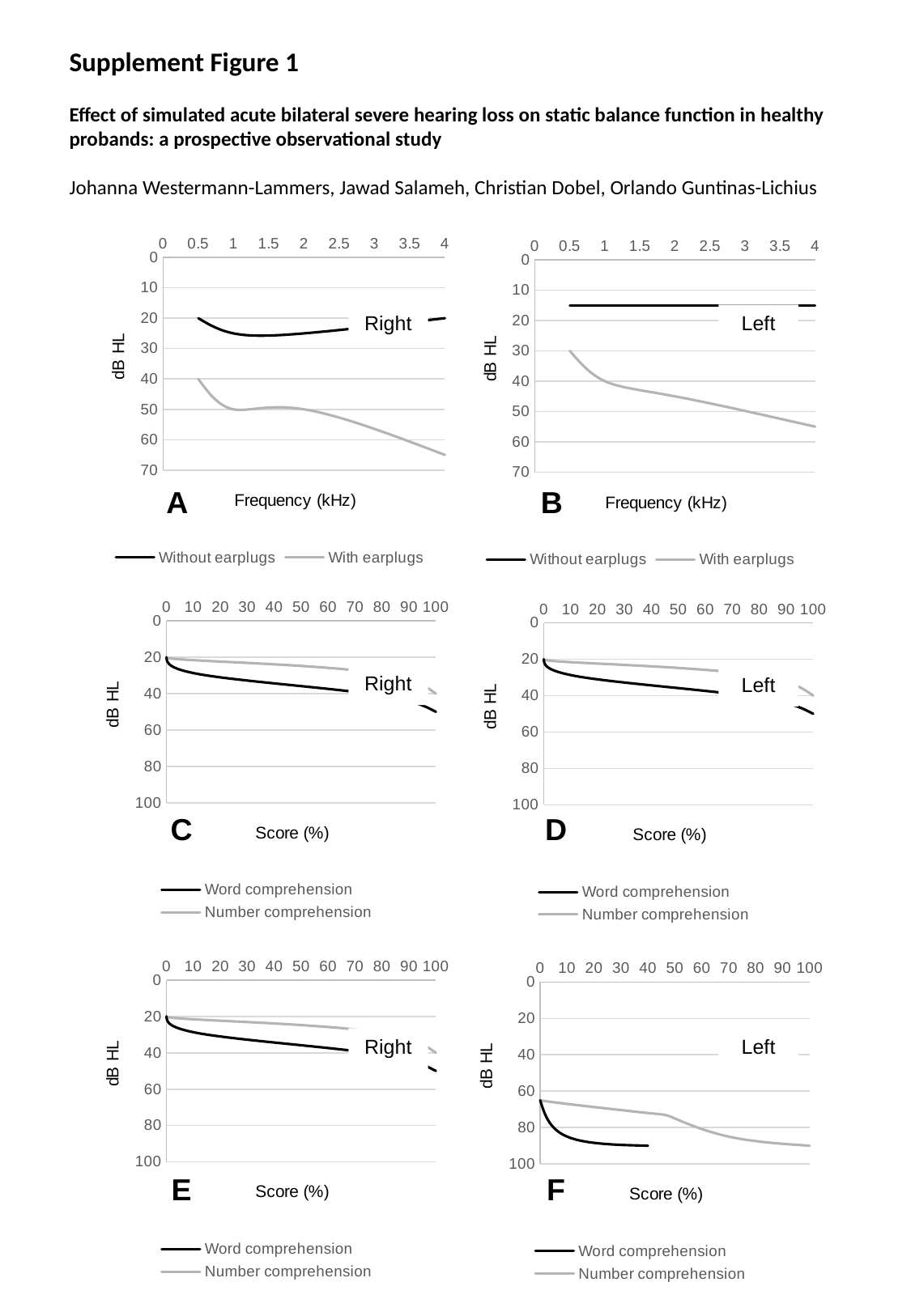

Supplement Figure 1
Effect of simulated acute bilateral severe hearing loss on static balance function in healthy probands: a prospective observational study
Johanna Westermann-Lammers, Jawad Salameh, Christian Dobel, Orlando Guntinas-Lichius
### Chart
| Category | Without earplugs | With earplugs |
|---|---|---|
### Chart
| Category | Without earplugs | With earplugs |
|---|---|---|Right
Left
A
B
### Chart
| Category | Word comprehension | Number comprehension |
|---|---|---|
### Chart
| Category | Word comprehension | Number comprehension |
|---|---|---|Right
Left
C
D
### Chart
| Category | Word comprehension | Number comprehension |
|---|---|---|
### Chart
| Category | Word comprehension | Number comprehension |
|---|---|---|Right
Left
E
F
